# Vaccenic acid suppresses intestinal inflammation by increasing anandamide and related *N*-acylethanolamines in the JCR:LA-cp rat[S]

**DOI:** 10.1194/jlr.M066308

**Published:** 2016-04

**Authors:** Miriam Jacome-Sosa, Claudia Vacca, Rabban Mangat, Abdoulaye Diane, Randy C. Nelson, Martin J. Reaney, Jianheng Shen, Jonathan M. Curtis, Donna F. Vine, Catherine J. Field, Miki Igarashi, Daniele Piomelli, Sebastiano Banni, Spencer D. Proctor

**Affiliations:** Metabolic and Cardiovascular Disease Laboratory,* Group on Molecular and Cell Biology of Lipids, Alberta Diabetes and Mazankowski Heart Institutes, University of Alberta, Edmonton, AB, Canada; Department of Biomedical Sciences,† University of Cagliari, Cittadella Universitaria, Monserrato, Cagliari, Italy; Department of Plant Science,§ University of Saskatchewan, Saskatoon, SK, Canada; Department of Agricultural Food and Nutritional Science,** University of Alberta, Edmonton, AB, Canada; Laboratory for Medical Homeostasis,†† RIKEN Center for Integrative Medical Sciences, Kanagawa, Japan; Departments of Anatomy and Neurobiology, Pharmacology, and Biological Chemistry,§§ University of California, Irvine, CA

**Keywords:** vaccenic acid, endocannabinoids, *N*-acylethanolamines, intestinal inflammatory diseases, anandamide

## Abstract

Vaccenic acid (VA), the predominant ruminant-derived *trans* fat in the food chain, ameliorates hyperlipidemia, yet mechanisms remain elusive. We investigated whether VA could influence tissue endocannabinoids (ECs) by altering the availability of their biosynthetic precursor, arachidonic acid (AA), in membrane phospholipids (PLs). JCR:LA*-cp* rats were assigned to a control diet with or without VA (1% w/w), *cis*-9, *trans*-11 conjugated linoleic acid (CLA) (1% w/w) or VA+CLA (1% + 0.5% w/w) for 8 weeks. VA reduced the EC, 2-arachidonoylglycerol (2-AG), in the liver and visceral adipose tissue (VAT) relative to control diet (*P* < 0.001), but did not change AA in tissue PLs. There was no additive effect of combining VA+CLA on 2-AG relative to VA alone (*P* > 0.05). Interestingly, VA increased jejunal concentrations of anandamide and those of the noncannabinoid signaling molecules, oleoylethanolamide and palmitoylethanolamide, relative to control diet (*P* < 0.05). This was consistent with a lower jejunal protein abundance (but not activity) of their degrading enzyme, fatty acid amide hydrolase, as well as the mRNA expression of TNFα and interleukin 1β (*P* < 0.05). The ability of VA to reduce 2-AG in the liver and VAT provides a potential mechanistic explanation to alleviate ectopic lipid accumulation. The opposing regulation of ECs and other noncannabinoid lipid signaling molecules by VA suggests an activation of benefit via the EC system in the intestine.

*Trans*-11 18:1 [vaccenic acid (VA)] is the most abundant ruminant-derived *trans* fatty acid (rTFA) in the food chain and has sparked major interest due to mandatory labeling of all *trans* fat on foods in North America. This interest has also been simulated by the recent announcement by the Food and Drug Administration to retract the generally recognized as safe (GRAS) status of “artificial” *trans* fatty acids (1). VA is also the precursor to endogenous synthesis of conjugated linoleic acid (CLA), the first rTFA to be recognized as having numerous health-related effects. Interestingly, a growing body of evidence from studies in animal models has suggested a bioactivity for VA independent of its conversion to CLA. More specifically, VA has been shown to attenuate complications observed in the metabolic syndrome, including dyslipidemia, fatty liver disease, and low-grade inflammation (2–5). It has been proposed that the lipid-lowering and anti-inflammatory effects of VA may be partially associated with its ability to ligand activate PPARγ-regulated pathways (6, 7) by acting directly in the intestine (6) and adipose tissue (5). In general, bioactive long chain fatty acids also act by modifying the composition of membrane phospholipids (PLs) and potentially replacing or interfering with the synthesis of PL-derived lipid signaling molecules, including endocannabinoids (ECs) (8, 9). However, the incorporation of VA into membrane PLs and potential effects on EC pathways remains unknown.

ECs, the endogenous ligands for cannabinoid (CB) receptors, are lipid-derived messengers that have emerged as key regulators of appetite behavior, energy metabolism, and intestinal inflammation. The most common ECs include arachidonoylethanolamide [anandamide (AEA)] and 2-arachidonoylglycerol (2-AG); and are derivatives of the PUFA, arachidonic acid (AA; C20:4, n-6), in PLs and can be modulated in response to dietary PUFA intake (10–13). Increased plasma EC concentrations, due to alterations in the activity/expression of enzymes regulating their biosynthesis and degradation, are associated with abdominal obesity, dyslipidemia, and insulin resistance in humans (14–16). Intriguingly, CB receptor over-activity can result in tissue-specific metabolic effects. For instance, central and hepatic CB receptor activation leads to hyperphagia (17), hepatic de novo lipogenesis, and insulin resistance (18–20). In contrast, in animal models of experimental colitis, increased CB receptor signaling has been shown to ameliorate smooth muscular irritation (21) and the T cell-mediated aberrant immune response (22); thus, counteracting the excessive inflammatory responses/signs in intestinal disease conditions. Therefore, nutritional interventions that target tissue-specific EC pathways could be used as potential therapeutic strategies for treatment of obesity-associated metabolic diseases.

A recent study in hypercholesterolemic subjects provided the first evidence that a dairy product naturally enriched with VA and CLA decreases plasma concentrations of AEA in a dose-dependent manner (23). However, the direct effect of VA on EC regulation was not able to be determined in this study. Furthermore, given the bioactive properties of VA to favorably modulate whole body energy metabolism and low-grade inflammation (5, 24), we proposed to explore novel regulatory effects of VA on tissue ECs as a potential mechanism of action for these metabolic effects. In order to address the specific role of dietary VA alone or in combination with CLA in EC metabolism, we supplemented the diets of an established rodent model of metabolic syndrome (the JCR:LA-*cp* rat) with these bioactive long chain fatty acids, and examined tissue concentrations of CB receptor ligands, AEA and 2-AG, and the biosynthetic precursor, AA, in membrane PLs. We also analyzed two noncannabinoid lipid signaling molecules, oleoylethanolamide (OEA) and palmitoylethanolamide (PEA), which only differ from AEA by their acyl chain and have been shown to induce satiety (25, 26) and exert anti-inflammatory effects through activation of the PPARα receptor (27).

## MATERIALS AND METHODS

### Animals and diets

Rats of the JCR:LA-*cp* strain that are homozygous for the corpulent trait (*cp/cp*) have a complete absence of the leptin receptor in the plasma membrane and spontaneously develop symptoms associated with the metabolic syndrome and the prediabetic state typically observed in humans; including obesity, insulin resistance, and dyslipidemia (28). Male JCR:LA-*cp* rats were raised in our established breeding colony at the University of Alberta, as previously described (29). At 8 weeks of age, rats (n = 5) were randomized and assigned to one of four diets (control and experimental diets) for 8 weeks and had free access to food and water.

The control diet was prepared by adding 1% cholesterol and 15% w/w of fat to an 85% basal mix diet (Harlan Laboratories; TD.06206) that contained 42% of energy from carbohydrate, 23.7% of energy from protein, and 34% of energy from fat, as previously described (5). Experimental diets were prepared by adjusting the fatty acid composition (replacing oleic acid with VA, CLA, or VA+CLA) of the control diet to provide approximately 1% w/w of VA (VA), 1% w/w of *cis*-9, *trans*-11 CLA (CLA), or both 1% w/w of VA + 0.5% w/w of *cis*-9, *trans*-11 CLA (VA+CLA). The amount of VA in the diet (∼2% of total energy from VA) was chosen based on previously published studies (2–4) and was intended to be compared with health effects of moderate doses of rTFA previously examined in human clinical trials (30). The fat composition of the control and experimental diets is shown in supplementary Table 1. Control and experimental diets were isocaloric and had a constant PUFA to SFA ratio of 0.4 and a constant n6 to n3 PUFA ratio of 8. Purified VA was synthesized by chemical alkali isomerization from linoleic acid-rich vegetable oil (31). Semi-purified *cis*-9, *trans*-11 CLA (G-c9t11 80:20) containing 59.8% of *cis*-9, *trans*-11 CLA and 14.4% of *trans*-10, *cis*-12 CLA was kindly provided by Lipid Nutrition. The fatty acid composition of diets was confirmed by gas chromatograph analysis (32) of the fat blend samples (**Table 1**). After euthanization, samples of the hypothalamus, skeletal muscle, visceral adipose tissue (VAT), liver, and jejunal mucosa segments of the intestine were excised and snap-frozen at −80°C until analysis. Animal care and experimental procedures were conducted in accordance with the Canadian Council on Animal Care and approved by the University of Alberta Animal Care and Use Committee-Livestock.

**TABLE 1.** Fatty acid composition of control and experimental diets

Fatty Acid	Control Diet	VA Diet	CLA Diet	VA+CLA Diet
C12:0	1.0	1.2	1.2	2.1
C14:0	3.6	4.2	4.4	7.6
C14:1	0.3	0.4	0.4	0.7
C16:0	19.0	18.1	17.7	22.6
C16:1	1.2	1.0	1.0	1.1
C18:0	11.0	10.9	10.6	7.7
C18:1 *trans*-9	0.5	0.5	0.4	0.2
C18:1 *trans*-11 (VA)	1.3	8.8	1.0	8.8
C18:1 *cis*-9 (oleic acid)	35.6	27.9	26.3	17.3
C18:1 *cis*-11	1.9	1.4	1.3	0.8
C18:2 n6	13.7	13.9	13.6	12.7
C18:3 n3	1.7	1.7	1.7	1.7
*cis*-9, *trans*-11 CLA	0.2	0.3	8.8	4.1
Summaries				
∑SFA	37.5	37.6	37.3	45.2
∑C12:0, C14:0, C16:0	23.6	23.5	23.3	32.2
PUFA	15.6	15.8	15.4	14.4
*cis*MUFA	40.0	31.6	29.8	20.5
*trans*MUFA	4.2	11.4	3.0	10.7
n6	13.8	13.8	13.8	13.8
n3	1.7	1.7	1.7	1.7
n6:n3 ratio	7.9	7.9	7.9	7.9
PUFA:SFA ratio	0.4	0.4	0.4	0.3

Values are expressed as percentage of fatty acids.

### Tissue lipid extraction

Tissues (0.2–0.3 g) were homogenized and total lipids extracted with chloroform/methanol (2:1, v/v) containing internal deuterated standards for AEA, 2-AG, OEA, and PEA to quantify for recovery efficiency ([^2^H]_8_-AEA, 20 ng/ml), ([^2^H]_5_-2-AG, 200 ng/ml), ([^2^H]_2_-OEA, 20 ng/ml), and ([^2^H]_4_-PEA, 20 ng/ml) (Cayman Chemical, Ann Arbor, MI). This mixture was washed with 0.25 vol of 0.9% KCl according to the Folch procedure (33) to separate the phases. Samples were centrifuged and the lipid-containing lower phase was transferred to clean tubes and evaporated to dryness under a stream of nitrogen at room temperature. After lipid extraction from tissue samples, lipid classes were separated by solid phase extraction using commercial silica cartridges, Strata SI-1 (Phenomenex, Torrance, CA). Samples were reconstituted in 500 μl of chloroform, vortexed, and loaded to the column followed by washing with 10 ml of chloroform to elute neutral lipids. The fractions containing ECs were then eluted with 10 ml chloroform/methanol (9:1, v/v), evaporated to dryness under nitrogen, and reconstituted in methanol until analysis by LC/MS. The fractions containing PLs were eluted with 10 ml methanol and stored at −20°C until further preparation for fatty acid analysis. Recovery of ECs in the chloroform/methanol (9:1, v/v) eluates was confirmed by LC/MS and estimated to be higher than 90%. Purity of the PL fraction was confirmed by TLC using heptane/isopropyl ether/acetic acid (60:40:4, by volume) as previously described (34).

### Analysis of ECs and AEA analogs

Samples were analyzed by LC-ESI-MS using an Agilent 1200 series HPLC coupled to a 3200 QTRAP mass spectrometer (AB SCIEX, Concord, ON, Canada). LC separation was performed through an Ascentis Express C18 column (7.5 cm × 2.1 mm, 2.7 μm particle size) at a flow rate of 0.3 ml/min. Two mobile phases were used: mobile phase A, methanol with 0.2% formic acid; and mobile phase B, 50 mM ammonium formate (pH 3). The gradient elution method started at 85% A from 0 to 0.1 min; then the mobile phase A linearly increased to 95% from 0.1 to 2 min and was held for an additional 2 min (from 2.1 to 4 min). Then, the mobile phase was returned to 85% A and was held at this composition for 6 min equilibrium time prior to the next injection. The mass spectrometer was operated in the multiple reaction monitoring scan mode under positive ionization. Nitrogen was used as curtain gas, for drying, and as nebulizing gas. AEA, 2-AG, and the two *N*-acylethanolamines (OEA and PEA) in their protonated forms [M+H]^+^ were identified as peaks with the appropriate *m/z* values and quantified by comparison with their external synthetic standards that were run under the same conditions. The multiple reaction monitoring transitions monitored were as follows: AEA *m/z* 348→62 (35 eV); AEA-d8 *m/z* 356→62 (35 eV); 2-AG *m/z* 379→287 (18 eV); 2-AG-d5 *m/z* 384→287 (18 eV); PEA *m/z* 300→62 (30 eV); PEA-d4 *m/z* 304→62 (30 eV); OEA *m/z* 326→62 (30 eV); OEA-d2 *m/z* 328→62 (30 eV). The linear range for the calibration (standard) curves for AEA, PEA, and OEA was 5–500 ng/ml and for 2-AG was 0.1–10 μg/ml. Because 2-AG and 1-AG undergo rapid isomerization (35), results for 2-AG were reported as the sum of the individual peaks of 2-AG + 1-AG.

### Fatty acid analysis in PLs

PL fractions were transesterified using 0.5 N methanolic base (metallic sodium in methanol) (Sigma-Aldrich) at 80°C for 15 min forming fatty acid methyl esters (FAMEs). The FAMEs were flushed with N_2_ and stored at −35°C until analysis and fatty acids were separated by GC with flame ionization detector (Varian 3900; Varian Inc., Mississauga, ON) using a 100 m CP-Sil 88 fused-silica capillary column [100 m × 0.25 mm i.d. × 0.2 μm film thickness (Varian Inc.)] as previously described (32). The FAMEs were identified by comparison with retention times of commercial GC reference FAME standards (FAME mix #463 and CLA FAME #UC-59M) from Nu-Chek Prep Inc.

### Measurement of fatty acid amide hydrolase protein expression in the jejunum

Samples of the jejunum were washed with phosphate-buffered saline, and a 2 cm segment was cut from 10 cm below the pyloric sphincter. Mucosal samples were then scraped from jejunal segments. Proteins from jejunal mucosa homogenates were separated by SDS-PAGE on Tris-acetate polyacrylamide gels (3–8%; Invitrogen), transferred to a polyvinylidene difluoride membrane, and incubated with anti-fatty acid amide hydrolase (FAAH)1 rabbit polyclonal antibody (1:1,000; catalog number 9179; Cell Signaling Technology®) and anti-β-actin mouse polyclonal antibody (1:5,000; catalog number ab8226; Abcam®, St Louis, MO), as previously described (36). Detection was achieved using anti-rabbit and anti-murine secondary antibodies and the ECL advance detection system (Amersham Biosciences). Results are expressed as a ratio of target protein:β-actin protein.

### Enzyme activity assays

The activity of FAAH and *N*-acylphosphatidylethanolamine phospholipase type D in jejunal mucosa was measured by standard assays, as previously described (37).

### Measurement of pro-inflammatory genes in the jejunum

Total RNA was isolated from frozen segments of jejunal mucosa using TRIzol® (Invitrogen, Canada), as described in the manufacturer’s protocol, and reversed transcribed into cDNA using MultiScribee reverse transcriptase (high-capacity cDNA reverse transcription kit; Applied Biosystems, Foster City, CA). The expression of CB1, FAAH, and the pro-inflammatory cytokines, TNFα and interleukin 1β (IL-1β) relative to the housekeeping gene, *Actb* (β-actin), was assessed by quantitative real-time PCR, using the StepOne™ Plus real-time PCR system (Applied Biosystems) and StepOne™ software (version 2). The PCR contained cDNA template, 100 nM of commercially available premixed target-specific primers, and TaqMan® FAM™-labeled probe (Applied Biosystems) for CB1, FAAH, TNFα, IL-1β, and *Actb*. Thermal cycling conditions were as follows: 95°C for 20 s, followed by 40 cycles of 95°C for 1 s and 60°C for 20 s. Relative mRNA expression for each target gene was normalized to *Actb* mRNA and quantified using the comparative cycle threshold (Ct) method. Data were expressed as the ratio of target mRNA expression relative to β-actin. All assays were performed in triplicate.

### Caco2 cell culture and measurement of FAAH inhibition on inflammatory cytokines

Caco2 cells (ATCC) were cultured in MEM (M4655; Sigma) with 10% fetal bovine serum and 1% penicillin/streptomycin and kept at 37°C in 5% CO_2_ and 95% humidity. Cells were grown in 6-well plates and seeded at 10^6^ cells per insert (24 mm diameter, 0.4 μm pore polycarbonate inserts). Cells at passages 15–25 were grown for a minimum of 18 days and used for experimentation between 18 and 21 days. Cells were maintained for 12 h in 1% fatty acid-free BSA after which they were treated with vehicle control, VA (100 μM), or VA (100 μM) + URB597 (FAAH inhibitor; Cedarlane) (1 μM) in the presence or absence of OEA (10 μM) for 24 h. Following this, the cells were challenged with lipopolysaccharide (LPS) (1 μg/μl) for 24 h. During this time, fresh vehicle control or VA (100 μM), URB597 (1 μM), OEA (10 μM) in 0.5% BSA was added. Total RNA was isolated from Caco2 cells and reversed transcribed into cDNA. The expression of TNFα relative to the housekeeping gene, GAPDH, was assessed by quantitative real-time PCR using SYBR Green (Applied Biosystems).

### Statistical analysis

All results are expressed as mean ± SEM. Statistical comparisons between dietary groups were analyzed using one-way ANOVA followed by Tukey’s post hoc test. The level of significance was set at *P* < 0.05 (Graph Pad Prism 5.0, USA).

## RESULTS

### Supplementation with VA alone or in combination with CLA does not affect EC concentrations in plasma, brain, or skeletal muscle

**Fig. 1.**
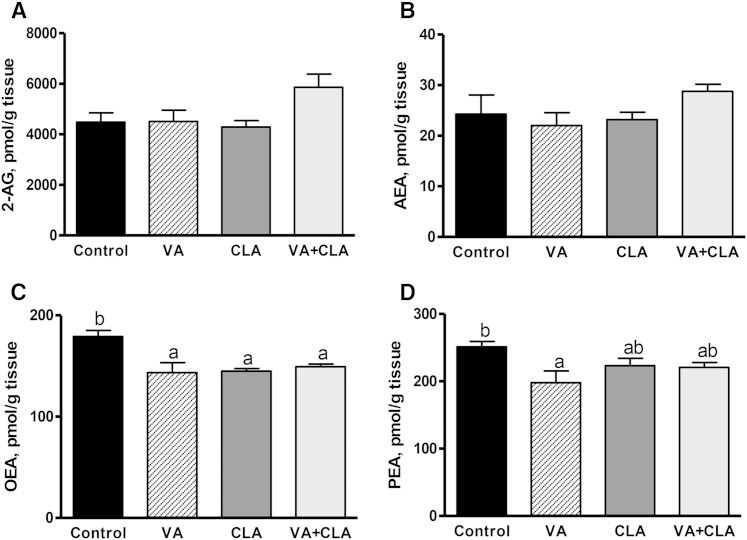
EC (A, B) and *N*-acylethanolamine (C, D) concentrations in the skeletal muscle of JCR:LA-*cp* rats following dietary supplementation with VA, CLA, or VA+CLA. Values are mean ± SEM, represented by vertical bars (n = 5). Means without a common letter differ (*P* < 0.05).

Contrary to our hypothesis, dietary supplementation with VA, CLA, or VA+CLA for 8 weeks did not affect EC concentrations in plasma (**Table 2**), the hypothalamus (data not shown), or skeletal muscle (**Fig. 1**) relative to control diet (*P* > 0.05). All dietary treatments (VA, CLA, and VA+CLA) significantly reduced (*P* < 0.01) the concentrations of OEA (−20, −19, and −17%, respectively) while only VA reduced the concentration of PEA (21%, *P* < 0.05) in the skeletal muscle relative to control (Fig. 1).

**TABLE 2.** EC and *N*-acylethanolamine concentrations in plasma of JCR:LA-*cp* rats fed control or experimental diets for 8 weeks

	Control		VA		CLA		VA+CLA	
	Mean	SEM	Mean	SEM	Mean	SEM	Mean	SEM
2-AG*^a^*	ND	—	ND	—	ND	—	ND	—
AEA*^a^*	8.9	4.1	5.6	0.6	7.9	0.4	6.4	1.9
PEA*^a^*	145.9	11.1	145.2	2.5	144.1	6.0	146.9	3.3
OEA*^a^*	62.1	6.8	59.3	1.8	59	6.5	57.9	3.1

Values are mean ± SEM, n = 3. Means did not differ, *P* > 0.05. ND, not detectable.

a Nanomoles per milliliter of plasma.

### Dietary supplementation with VA alone or in combination with CLA decreases the concentration of 2-AG and AEA analogs (OEA and PEA) in the liver and VAT

**Fig. 2.**
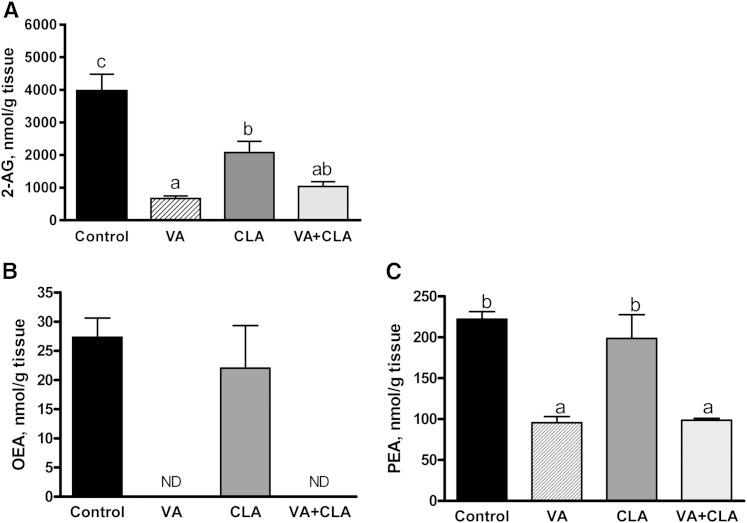
EC (A) and *N*-acylethanolamine (B, C) concentrations in the liver of JCR:LA-*cp* rats following dietary supplementation with VA, CLA, or VA+CLA. Values are mean ± SEM, represented by vertical bars (n = 5). Means without a common letter differ (*P* < 0.05). ND, not detectable

Supplementation with VA, CLA, or VA+CLA significantly reduced 2-AG concentrations in the liver (83%, *P* < 0.001; 47.6%, *P* < 0.05; and 74%, *P* < 0.001, respectively) relative to control diet. Interestingly, the VA diet (but not the VA+CLA diet) lowered liver 2-AG concentrations by 68% as compared with the CLA-fed group (*P* < 0.05). In addition, VA or VA+CLA supplementation (but not CLA) resulted in decreased (*P* < 0.001) concentrations of the *N*-acylethanolamines, OEA (to not detectable levels) and PEA (−57 and −56%, respectively) in this tissue as compared with control (**Fig. 2**). AEA was not detected in the liver of JCR:LA-*cp* rats.

**Fig. 3.**
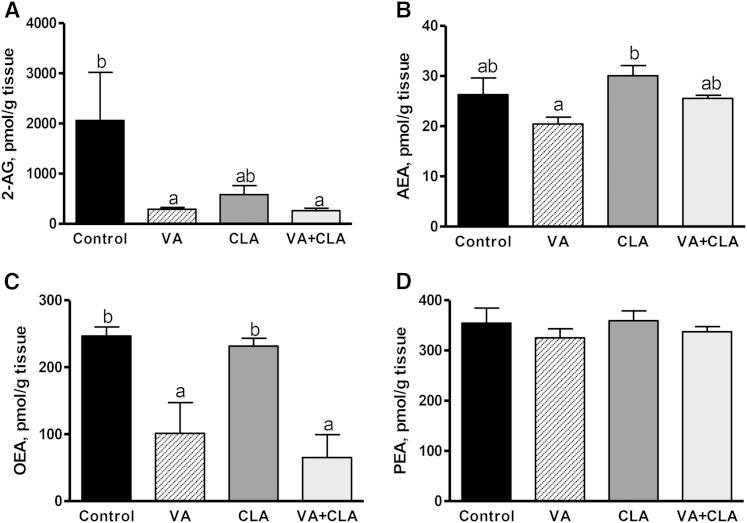
EC (A, B) and *N*-acylethanolamine (C, D) concentrations in VAT of JCR:LA-*cp* rats following dietary supplementation with VA, CLA, or VA+CLA. Values are mean ± SEM, represented by vertical bars (n = 5). Means without a common letter differ (*P* < 0.05).

VA and VA+CLA (but not CLA) significantly (*P* < 0.05) reduced the concentrations of 2-AG (−86 and −87%, respectively) and OEA (−59%, *P* < 0.05 and −74%, *P* < 0.01, respectively) in VAT as compared with control (**Fig. 3**).

Collectively, results from this study suggest that there were no additive effects of combining VA with CLA on reducing EC concentrations in liver and VAT.

### Supplementation with VA increases the concentration of AEA and AEA analogs (OEA and PEA) in the jejunum

**Fig. 4.**
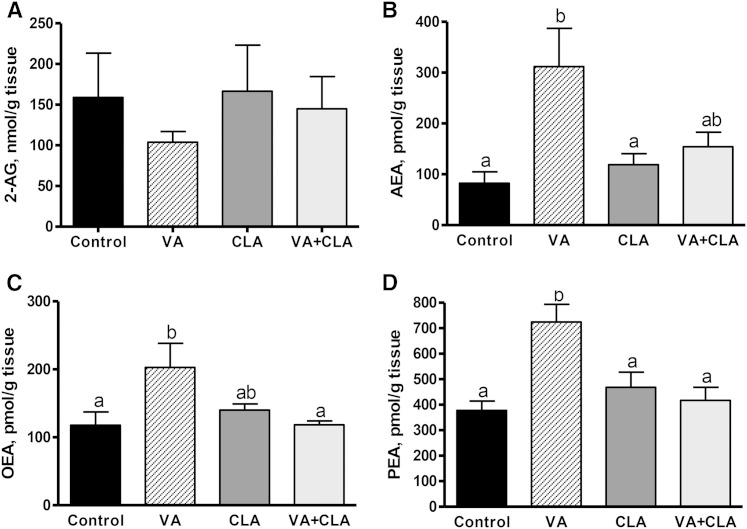
EC (A, B) and *N*-acylethanolamine (C, D) concentrations in the jejunal mucosa of JCR:LA-*cp* rats following dietary supplementation with VA, CLA, or VA+CLA. Values are means ± SEM, represented by vertical bars (n = 5). Means without a common letter differ (*P* < 0.05).

Unexpectedly, dietary supplementation with VA (but not CLA or VA+CLA) significantly increased jejunal concentrations of the EC, AEA (3.8-fold, *P* < 0.05), and its analogs, OEA (1.7-fold, *P* < 0.05) and PEA (1.9-fold, *P* < 0.01) (**Fig. 4**). It is important to note that VA did not alter food intake in the present study (data not shown) or in previous studies using different animal models (2, 3, 38, 39). Therefore, it is unlikely that increased AEA concentrations in the jejunum (following VA treatment) would result in an appetite stimulatory effect as a result of CB receptor activation.

### Effects of dietary supplementation with VA, CLA, or their combination on tissue PL fatty acid composition

We analyzed tissue PL fatty acid composition to determine whether changes in tissue EC and *N*-acylethanolamine concentrations were due to alterations in the availability of their biosynthetic precursors in membrane PLs (**Table 3**). Interestingly, we found that VA supplementation increased the EC precursor, AA, in liver (30%, *P* < 0.001) and skeletal muscle (11%, *P* < 0.05) PLs compared with control diet. VA+CLA-fed rats also had increased amounts of AA (20%) in liver PLs relative to control rats (*P* < 0.01). The incorporation of AA in VAT, jejunal, and hypothalamus PLs was not affected by any experimental diet (VA, CLA, or VA+CLA) relative to control (*P* > 0.05). VA- and VA+CLA-fed rats had lower concentrations of the OEA precursor, oleic acid (−35 and −40%, respectively), while VA-fed rats only had lower amounts of the PEA precursor, palmitic acid (−21%), in liver PLs relative to control rats (*P* < 0.001). CLA- and VA+CLA-fed rats had lower (*P* < 0.001) amounts of oleic acid (−15 and −17%, respectively) in VAT PLs, while only VA+CLA rats had lower concentrations of oleic acid (−33%, *P* < 0.01) in jejunal PLs relative to control rats. Collectively, findings from fatty acid analysis in tissue PLs suggest that the regulatory effect of VA on tissue ECs could not be explained by changes in their biosynthetic membrane PL precursor (AA).

**TABLE 3.** PL fatty acid composition in tissues of JCR:LA-*cp* rats fed control or experimental diets for 8 weeks

	Control		VA		CLA		VA+CLA	
Fatty Acids*^a^*	Mean	SEM	Mean	SEM	Mean	SEM	Mean	SEM
Liver								
16:0	16.4^b^	0.7	12.9^a^	0.3	15.7^b^	0.4	14.6^ab^	0.5
*cis*-9 18:1 (OA)	6.0^b^	0.3	3.9^a^	0.1	5.2^b^	0.2	3.6^a^	0.1
*trans*-11-VA	0.2^a^	0.1	1.6^c^	0.0	0.7^b^	0.0	1.6^c^	0.1
*cis*-9, *trans*-11 CLA	ND	—	ND	—	0.6^b^	0.0	0.3^a^	0.0
20:4 *n*6 (AA)	19.7^a^	0.7	25.2^b^	0.5	18.5^a^	0.7	24.2^b^	0.6
VAT								
16:0	10.8	1.2	9.5	0.1	11.3	1.3	10.3	0.1
*cis*-9 18:1 (OA)	11.8^b^	0.3	11.6^b^	0.2	10.0^a^	0.2	9.8^a^	0.2
*trans*-11 VA	0.4^a^	0.1	1.8^c^	0.0	0.7^b^	0.1	1.9^c^	0.0
*cis*-9, *trans*-11 CLA	ND	—	ND	—	0.8^b^	0.1	0.6^a^	0.0
20:4 *n*6 (AA)	15.0	1.3	13.9	0.2	14.5	0.9	14.1	0.3
Hypothalamus								
16:0	17.6	0.3	17.1	0.3	17.2	0.4	18.2	0.4
*cis*-9 18:1 (OA)	21.4	0.3	21.9	0.2	21.0	0.7	20.7	0.6
*trans*-11 VA)	ND	—	0.1^b^	0.0	0.04^a^	0.0	0.1^b^	0.0
*cis*-9, *trans*-11 CLA	ND	—	ND	0.0	0.1^c^	0.0	0.08^b^	0.0
20:4 *n*6 (AA)	9.9	0.2	9.6	0.2	10.2	0.5	10.3	0.6
Muscle								
16:0	19.8	1.9	21.3	0.7	21.0	0.6	17.1	0.8
*cis*-9 18:1 (OA)	5.3	0.4	5.3	0.4	5.7	0.4	4.6	0.4
*trans*11 VA	0.1^a^	0.1	1.0^b^	0.0	0.1^a^	0.1	1.2^b^	0.1
*cis*-9, *trans*-11 CLA	ND	—	ND	—	ND	—	ND	—
20:4 *n*6 (AA)	11.7^a^	0.3	13.0^b^	0.3	11.8^ab^	0.4	10.8^a^	0.3
Jejunal mucosa								
16:0	13.2	0.5	11.6	0.3	13.0	0.3	12.5	0.8
*cis*-9 18:1 (OA)	8.3^b^	0.1	7.2^ab^	0.4	7.7^b^	0.6	5.6^a^	0.4
*trans*-11 VA	0.4^a^	0.0	1.5^c^	0.1	0.6^b^	0.1	1.4^c^	0.1
*cis*-9, *trans*-11 CLA	ND	—	ND	—	0.5^b^	0.0	0.2^a^	0.0
20:4 *n*6 (AA)	14.5	0.6	18.3	1.5	14.7	1.5	20.0	1.6

Values are mean ± SEM, n = 5. Means in a row with superscripts without a common letter differ, *P* < 0.05. OA, oleic acid; ND, not detectable.

a Grams per 100 grams total fatty acids.

We also assessed the PL incorporation of VA and CLA in all tissues analyzed. As expected, VA- and VA+CLA-fed rats had higher (*P* < 0.001) concentrations of VA in liver (8-fold), VAT (4.5-fold), skeletal muscle (10-fold), and jejunal (3.8-fold) PLs compared with rats fed the control diet. The incorporation of VA into the hypothalamus of JCR:LA-*cp* rats was found to be at trace amounts (0.1 g/100 g total fatty acids).

CLA was not detected in tissues of VA-fed rats, suggesting limited incorporation of CLA (produced from VA desaturation) into tissue PLs. Furthermore, CLA was only incorporated in the liver, VAT, hypothalamus, and jejunal PLs of CLA-fed rats (<1 g/100 g total fatty acids) and to a lesser extent in tissue PLs of rats fed the VA+CLA diet (*P* < 0.05).

### The magnitude of incorporation of VA in membrane PLs is tissue dependent

**Fig. 5.**
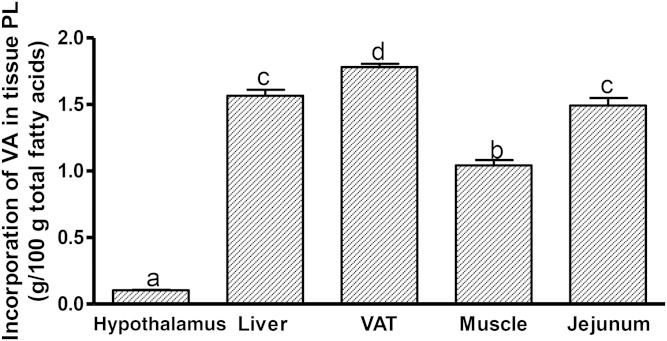
Incorporation of VA in tissue PLs in JCR:LA-*cp* rats supplemented with VA for 8 weeks. Values are mean ± SEM, represented by vertical bars (n = 5). Means without a common letter differ, *P* < 0.05.

To associate the tissue-specific effects of VA with its magnitude of incorporation, we conducted a comparison of VA in tissue membrane PLs (**Fig. 5**). VA was incorporated in VAT (1.8 g/100 g fatty acids), followed by the liver (1.6 g/100 g fatty acids), jejunum (1.5 g/100 g fatty acids), skeletal muscle (1.0 g/100 g fatty acids), and hypothalamus (0.1 g/100 g fatty acids).

### Dietary supplementation with VA alters the mRNA and protein expression of FAAH, but does not affect the mRNA expression of CB1 receptor in the jejunum

**Fig. 6.**
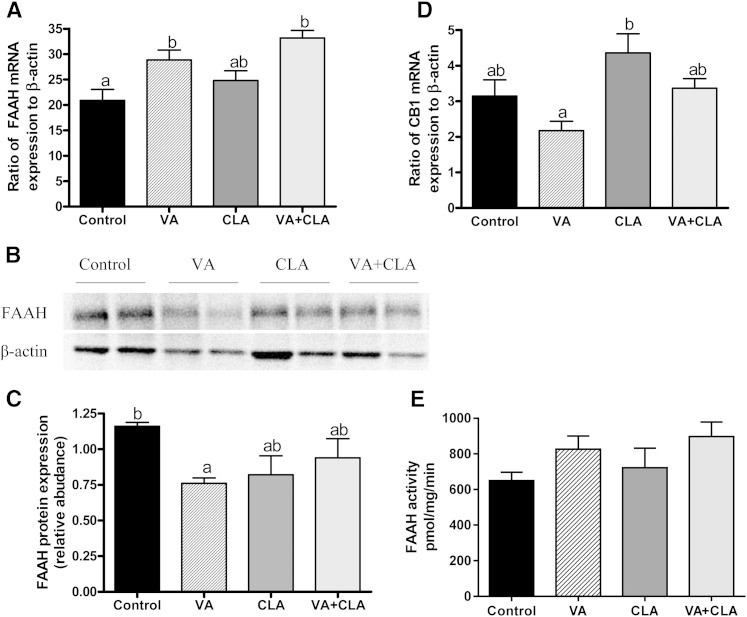
Jejunal mucosal mRNA expression of FAAH and CB1 and protein abundance and activity of FAAH in JCR:LA-*cp* rats fed control or experimental diets for 8 weeks. The mRNA expression for FAAH (A) and CB1 (D) is relative to the housekeeping gene, β-actin. A representative blot (B) and graph (C) for FAAH relative protein abundance are shown. FAAH activity (E) was not significant within groups. Values are mean ± SEM, represented by vertical bars (n = 5). Means without a common letter differ, *P* < 0.05.

To determine whether selective increase of AEA, OEA, and PEA in the jejunal mucosa by VA could be associated with synthetic or degradative pathways, we first measured the mRNA expression and protein activity of jejunal *N*-acylphosphatidylethanolamine phospholipase type D, one of the key enzymes responsible for the synthesis of these *N*-acylethanolamines, but no differences were found between groups (data not shown, *P* > 0.05). We then measured the expression of FAAH, their primary hydrolyzing enzyme. Interestingly, while VA- and VA+CLA-fed rats had higher jejunal mRNA expression of FAAH compared with the control group (1.4-fold, *P* < 0.05 and 1.6-fold, *P* < 0.01, respectively), the protein abundance of this enzyme was reduced in VA-fed rats only (−34%, *P* < 0.05) (**Fig. 6A–C**). However, the protein activity of jejunal FAAH did not differ between groups (Fig. 6E, *P* > 0.05). We also found that VA tended to lower jejunal mRNA expression of CB1 relative to the other diets; however, this did not reach statistical significance (*P* > 0.05) (Fig. 6D).

### Dietary supplementation with VA reduced the mRNA expression of pro-inflammatory cytokines in the jejunum

**Fig. 7.**
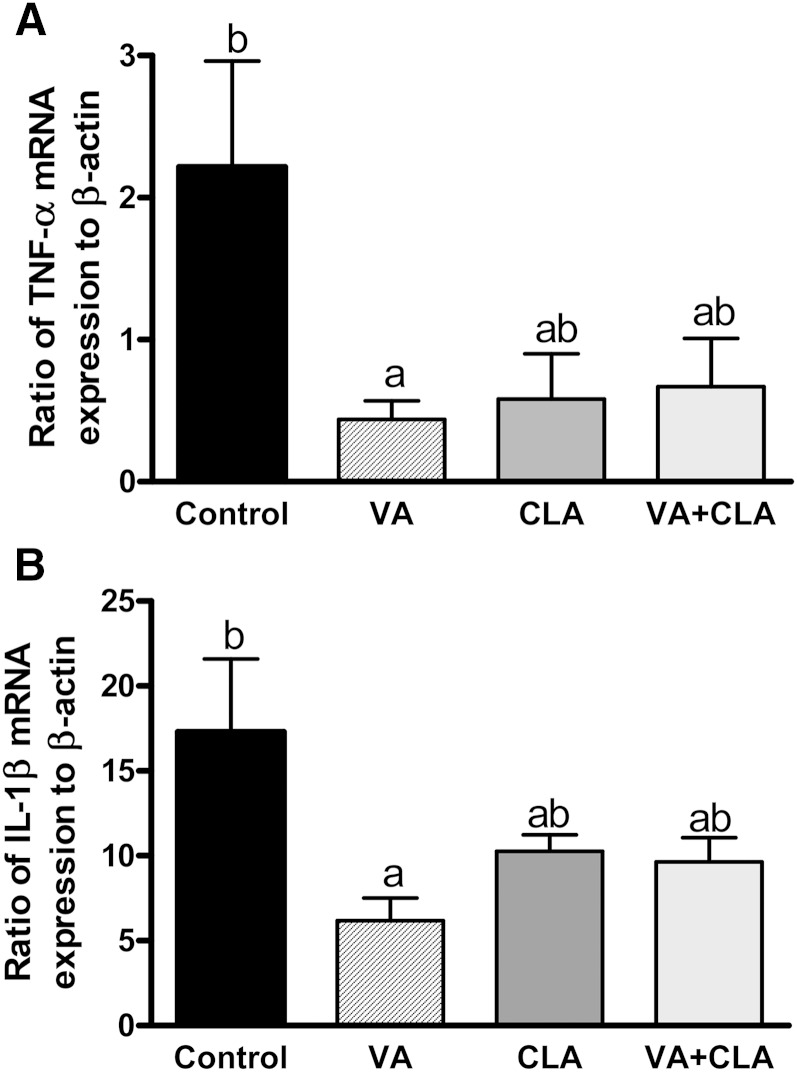
Jejunal mucosa mRNA expression of pro-inflammatory cytokines in JCR:LA-*cp* rats fed control or experimental diets for 8 weeks. The expression of TNFα (A) and IL-1β (B) is relative to the housekeeping gene, β-actin. Values are mean ± SEM, represented by vertical bars (n = 5). Means without a common letter differ, *P* < 0.05.

The EC system (ECS) is upregulated in human inflammatory bowel diseases and experimental models of colitis and colorectal cancer growth (21, 40–42). During these conditions, an overactive ECS is proposed to be an adaptive response to counteract the consequences of inflammation, such as T cell-mediated aberrant immune response (41). Therefore, we explored whether the increase in jejunal AEA, OEA, and PEA that we observed was associated with intestinal inflammation. Indeed, the expression of pro-inflammatory cytokines [TNFα (**Fig. 7A**) and IL-1β (Fig. 7B)] in the intestine was significantly lower in rats fed the VA-supplemented diet compared with the control rats (−80 and −64%, respectively; *P* < 0.05).

### Caco2 cell culture to verify VA action on anti-inflammatory pathways

**Fig. 8.**
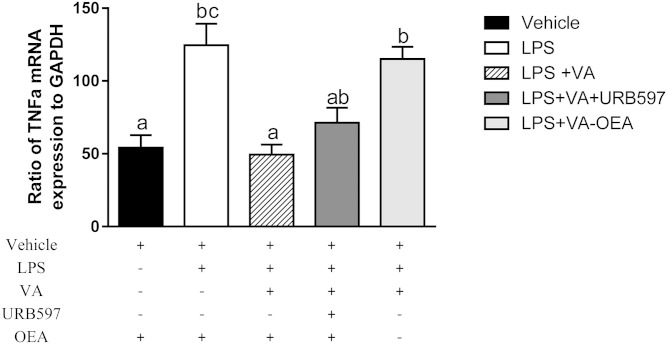
Effect of VA in the presence or absence of OEA and the FAAH inhibitor, URB597, in the intestinal Caco2 cell model of inflammation. The expression of TNFα is relative to the housekeeping gene, GAPDH. Values are mean ± SEM, represented by vertical bars (n = 5). Means without a common letter differ, *P* < 0.05.

We tested the anti-inflammatory properties of VA alone or in combination with the FAAH inhibitor, URB597, in the presence or absence of the FAAH substrate, OEA, in the Caco2 cell model of human intestinal epithelial cells. Treatment with VA alone reduced the LPS-induced mRNA expression of TNFα by 64%, while addition of URB597 did not result in reduction in Caco2 cells (**Fig. 8**). The mRNA expression of TNFα was not reduced by VA in the absence of OEA (*P* > 0.05), suggesting that OEA is required for the observed anti-inflammatory properties of VA in this cell model.

## DISCUSSION

### Dietary supplementation with VA reduces liver and VAT 2-AG without altering the availability of PL biosynthetic precursor

Increased plasma EC concentrations have been found to be positively correlated with visceral fat mass and waist circumference in humans (14–16). Consequently, the ECS has been proposed as a critical target for the treatment of abdominal obesity and associated metabolic abnormalities in the metabolic syndrome. While pharmacological blockade of the CB1 receptor with rimonabant has shown some clinical success, the adverse psychiatric side effects associated with this drug have led to its withdrawal as a treatment option (43). Given that the ECS can be modulated in response to dietary fat (9), nutritional interventions with tissue-specific effects could be an attractive alternative approach to clinically target the systemic ECS during metabolically abnormal conditions.

To our knowledge, this is the first report to demonstrate that despite a lack of a direct effect on either the plasma or the brain ECs, dietary supplementation with VA can effectively decrease liver and VAT 2-AG concentrations in a rat model of metabolic syndrome. Our findings resemble those reported for n3 long chain PUFA (in the form of krill oil) in the Zucker *fa/fat* rat (44) and may also suggest a putative mechanism of action for the ability of VA to decrease ectopic lipid accumulation and hepatic TG secretion (5). The rationale for this hypothesis is that CB receptor activation in the liver and VAT can lead to increased de novo lipogenesis and visceral adiposity (18, 19, 45), both of which are attenuated by VA (3, 5). Interestingly, effects of VA on liver and VAT 2-AG concentrations cannot be explained by changes in AA levels in membrane PLs, but rather due to incorporation of VA into PLs. Thus, it is plausible that an increased incorporation of VA (relative to AA) into the lipid precursor of 2-AG (i.e., diacylglycerol) may occur during PL remodeling. This modification would result in the synthesis of a VA-derived glycerol compound instead of synthesis of 2-AG, thereby leading to decreased concentrations of 2-AG, but this requires further investigation.

### Effects of VA on tissue EC concentrations are associated with its incorporation into tissue PLs

Previous studies in the JCR:LA-*cp* rat have shown that the lipid-lowering effects of CLA are enhanced by the addition of VA when compared with dietary supplementation with CLA alone (24). In this study, we provide evidence that VA per se can independently reduce tissue 2-AG concentrations corresponding with its magnitude of incorporation into tissue PLs when compared with CLA (Table 3). Our findings also reveal that effects of VA on EC concentrations may be tissue-specific and parallel the extent of VA incorporation into respective tissue membrane PLs (Fig. 5). We note that while the incorporation of VA in peripheral tissues (VAT, liver, and jejunal mucosa) is within the same general level, the lower hypothalamic incorporation of VA suggests a decreased active transport of VA across the blood brain barrier, but this requires further investigation. Further studies are also needed to determine the mechanism of how the incorporation of VA into tissue PLs mediates a lowering of EC concentrations in liver and VAT.

### Increased jejunal AEA, OEA, and PEA by VA is associated with downregulation of FAAH protein expression and may explain the anti-inflammatory properties of VA

We have observed that VA re-equilibrates intestinal and hepatic lipid homeostasis while exerting differential transcriptional regulation in both organs, as reflected in mRNA levels of sterol regulatory element-binding protein 1 (SREBP1) and FAS (5). In this study, we found a consistent effect of VA to decrease 2-AG concentrations in the liver and VAT. In contrast, jejunal concentrations of AEA and its analogs (OEA and PEA) were selectively increased following VA treatment alone. This selective increase of jejunal *N*-acylethanolamines by VA could not be explained by changes in their biosynthetic PL precursors, but associated with a reduction in protein expression of the enzyme, FAAH (known to hydrolyze AEA, OEA, and PEA). Notably, feeding n3 long chain PUFAs is associated with an increase in the FAAH inhibitor, arachidonoyl-serotonin (AA-5-HT), and other jejunal long chain PUFA-serotonins (also capable of inhibiting FAAH activity in vitro) in mice (46). Interestingly, in the present study, we found discordance between increased mRNA levels and reduced protein abundance of FAAH in the jejunal mucosa upon VA supplementation, which suggests a compensatory mechanism for the reduced protein and infers an active feedback pathway for the enzyme. Furthermore, the protein activity of FAAH was not different between groups (Fig. 6E). Although it is plausible that VA supplementation may stimulate the formation of lipid mediators that regulate FAAH abundance, the factors that regulate FAAH abundance and/or activity are complex. For example, the literature describes a complex interaction of FAAH with appetite and sex hormones (47, 48), as well as preferential membrane stabilization of FAAH by sterols (49) that could potentially be involved.

Targeting the ECS (via inhibition of FAAH) has therapeutic potential in the treatment of intestinal inflammatory diseases (50–52). AEA is found to be increased in human intestinal inflammatory diseases and is proposed to be an adaptive response to counteract the inflammatory milieu during these conditions (40–42). In contrast to the pathological role of the ECS in the liver and VAT (13, 18, 53), activation of CB1 or CB2 receptors (via exogenous agonists or FAAH inhibition) has been shown to reduce macroscopic damage scores of colonic inflammation and to attenuate the activation of T-cells and the infiltration of inflammatory cells in animal models of colitis (21, 22, 50, 51). Additionally, the *N*-acylethanolamine PEA has also been shown to exert anti-inflammatory effects through activation of PPARα, including reduction of pro-inflammatory cytokine production (54). We therefore examined the mRNA expression of two key inflammatory markers, TNFα and IL-1β, in the intestine to explore a potential association with the increased AEA and PEA concentrations observed in the jejunum. Indeed, the reduction in mRNA expression of these pro-inflammatory cytokines by VA is consistent with previous observations in this rat model. VA treatment has been shown to normalize the production of IL-2 and TNFα in mesenteric gut-associated lymph nodes after mitogen stimulation in this rodent model of metabolic syndrome (4). Results from the present study provide further evidence for a potential anti-inflammatory effect of VA via activation of the ECS in the intestine. We wish to note that both CB and noncannabinoid receptor (e.g., PPAR) mechanisms may contribute to the anti-inflammatory effects of FAAH inhibition (52). Further, we have shown previously that VA feeding increases the expression of PPARα and PPARγ in jejunal mucosa (6). Therefore, increasing the availability of endogenous ligands to CB and PPAR receptors (e.g., AEA and PEA, respectively) may offer a mechanistic explanation for the anti-inflammatory effects of VA in this organ.

Results from this study also suggest a potential synergistic effect of VA and OEA on reducing the LPS-induced mRNA expression of TNFα in the Caco2 cell model (Fig. 8). The mechanisms for this interaction are not clear, but previous studies suggest that the anti-inflammatory effects of OEA are associated with reduced activation of the transcriptional cytokine activator, nuclear factor κB (NF-κB) (55, 56). Unlike industrially derived *trans* fatty acids, VA has been shown to protect against NF-κB activation (57) and inflammation, as measured by reduced T-helper cell cytokine production in a PPARy-dependent manner (7). Therefore, it is plausible that OEA and VA may act synergistically to reduce the LPS-induced pro-inflammatory response via PPAR activation. Alternatively, VA and OEA may alter cell receptor function (e.g., toll-like receptor 4) and receptor-induced activation of NF-κB indirectly by altering membrane and lipid raft function, but this warrants further investigation.

In conclusion, we demonstrate that dietary supplementation with VA exerts a tissue-specific regulation of ECs that could be used as an attractive alternative approach to target the ECS during conditions of metabolic syndrome and intestinal inflammatory diseases. We have shown that VA effectively reduces liver and VAT 2-AG concentrations corresponding with its previously observed properties to beneficially modulate lipid storage compartments. We have also provided evidence that VA can act independently of CLA, which seems to be associated with its incorporation into tissue PLs. Additionally, the present findings delineate a unique opposing regulation of VA on AEA and its *N*-acylethanolamine analogs that cannot be explained by changes in their biosynthetic PL precursors. Rather, our results suggest an inhibitory effect of VA on the protein expression of FAAH in the intestine that may result in activation of protective pathways of the ECS in this organ. Collectively, findings from this study have provided a potential novel mechanism of action for the health benefits of VA and highlight the need for further investigation to explore the efficacy of VA on intestinal inflammatory diseases.

## Supplementary Material

Supplemental Data
