# Supplementary material for: Vaccenic acid suppresses intestinal inflammation by increasing anandamide and related N-acylethanolamines in the JCR:LA-cp rat
Source: J Lipid Res. 2016 Apr;57(4):638–49. doi: 10.1194/jlr.M066308 (PMC4808772; doi:10.1194/jlr.M066308)
Supplement: Supplemental Data [file supp_57_4_638__index.html]

Vaccenic acid suppresses intestinal inflammation by increasing the endocannabinoid anandamide and non-cannabinoid signaling molecules in a rat model of the metabolic syndrome. — Vaccenic acid suppresses intestinal inflammation by increasing anandamide and related N-acylethanolamines in the JCR:LA-cp rat — Supplemental Data 

# Vaccenic acid suppresses intestinal inflammation by increasing anandamide and related *N*-acylethanolamines in the JCR:LA-cp rat

## Supplemental Data

- Supplemental Table 1 (.pdf, 88 KB) - Supplemental Table 1 Fat composition of designed control and experimental diets
