# Supplementary material for: Vaccenic acid suppresses intestinal inflammation by increasing anandamide and related N-acylethanolamines in the JCR:LA-cp rat
Source: J Lipid Res. 2016 Apr;57(4):638–49. doi: 10.1194/jlr.M066308 (PMC4808772; doi:10.1194/jlr.M066308)
Supplement: Supplemental Data [file 10.1194_M066308_jlr.M066308-1.pdf]

Supplemental Table 1 Fat composition of designed control and experimental diets

Fat ingredient	Control diet	VA diet	CLA diet	VA+CLA diet
	g/kg of diet			
Butter	48.75	57.3	59.6	102.50
Sunflower oil	14.25	21.6	22.5	0
Safflower oil	0	0	0	22.13
Flaxseed	3.75	3.9	3.9	3.9
Fully hydrogenated canola oil	6.75	6.6	6.75	0
Olive oil	52.5	28.5	25.5	0
Lard	24	19.4	13.5	0
Vaccenic acid	0	12.66	0	13.82
Conjugated linoleic acid	0	0	18.2	8.25

Values are expressed as g per kg of diet. Adapted from reference 5.
